# Measuring body fat—How accurate is the extrapolation of predictive models in epidemiology?

**DOI:** 10.1371/journal.pone.0263590

**Published:** 2022-02-10

**Authors:** Jean-Claude Pineau, Fernando V. Ramirez Rozzi

**Affiliations:** 1 CNRS, BABEL, Université de Paris, Paris, France; 2 UMR 7206 Ecoanthropology, MNHN, CNRS, UP, Paris, France; 3 UR 2496, Faculté de Chirurgie Dentaire, UP, Montrouge, France; Tabriz University of Medical Sciences, ISLAMIC REPUBLIC OF IRAN

## Abstract

Excess fat is a risk factor for many chronic diseases which can lead to premature mortality. Many studies have proposed predictive equations for body fat mass and body fat mass percentage based on anthropometric measures in relation to age and sex. However, the use of these predictive equations on other subject samples may not be relevant. Our objective is to assess whether the predictive equations proposed in the literature are generalizable to any population. We obtained fat mass and fat percentage on a reference population using Absorptiometry DXA. The predictive equations were applied to our population and the mean and individual differences between actual and estimated values were obtained. Predictive equations obtained from a reduced number of subjects have a very high Standard Error of Estimate (>3) and therefore their accuracy is not acceptable. Only the formulae established from a large number of individuals allow the estimation of values whose Standard Error of Estimate is less than 3. These equations, thanks to the large sample size, include a sufficiently large variability in anthropometric measurements covering the diversity of anthropometric values for the same fat value. However, predictive equations based on a large sample size, while exhibiting no current difference in variances, can show a shift in mean values. This mean-shift is the result of differences in DXA devices and needs to be corrected. It means that DXA values from a few individuals in the population under study must be obtained to calculate a corrective factor.

## Introduction

Excess body fat is a risk factor for many chronic diseases which can lead to premature mortality [1–3]. Global obesity has nearly doubled in the last three decades [4]. In France, the prevalence of obesity among adults is 11.3%, or about 3.5 million people. Obesity is often associated with serious cardiovascular and health risks [5]. Preventing excess body fat has become a primary objective in epidemiological studies.

Absorptiometry (DXA) is a recognized reference method for measuring body composition in cross-sectional and longitudinal studies [6, 7]. The DXA technique scans the whole body with an X-ray beam at two energy levels (70 and 100 Kev) and is a reference method for measuring fat, lean mass and mineral content. However, this technique has the disadvantage of radiation exposure with relatively high cost and limited accessibility. For this reason, many studies have proposed body fat mass (BF) and body fat mass percentage (BF%) predictive equations established using anthropometric measurements in relation to age and sex. The results obtained by anthropometric measurements and those obtained by DXA absorptiometry are compared and the effectiveness of the equations is measured by the values of the R^2^ (coefficient of determination) and the SEE (Standard Error of Estimate) [8–13]. This procedure was used in studies comprising small [14, 15] and very large cohorts of subjects [12, 13]. In practice, most authors performed validations of their equations on samples external to their population that served as a model. However, the use of these predictive equations on other samples of subjects with a greater diversity of measurements of the selected variables may not be relevant. Indeed, we know that for the same total BF or the same BF% there may be a significant variability of anthropometric dimensions depending on the samples concerned and therefore a greater variability will inevitably lead to a less precise prediction. It is to be expected that equations obtained from studies on very large cohorts of individuals are more accurate as they consider the variability of anthropological measurements for every measurement of body fat.

It is worth noting that most studies comparing body composition measurements with DXA values lead to different equations. This is primarily due to the use of different DXA devices because each device differs in terms of calibration, software and scan speed [16, 17]. Lantz et al. [18] have suggested that standardization of DXA devices has become essential because of the very large differences in their results.

The objective of this study is to assess whether the BF and BF% predictive equations proposed in the literature are generalizable to any population taking into account the sample size on which the equation was derived and the use of different DXA devices. In order to do this anthropometric measurements used in predictive equations as well as BF and BF% by DXA were obtained from a reference population. The mean and individual differences between BF and BF% by DXA and the results of different equations were obtained, and the effectiveness of the equations assessed by SEE.

## Materials and methods

This study, was carried out in a university teaching hospital (CHU) at Angers in France, under a French medical agreement. The measurements were taken as part of a European « Body Life » program in 2001–2003. Data collection was carried out for a period of 6 months. A sample of 120 men was recruited from hospital consultations or hospitalization units. The majority of the subjects were Caucasians (95%). The l8-82-year-old patients were recruited according to a wide range of body mass index (BMI) and consequently of fat mass. 30% of the patients had a BMI >30 kg/m^2^, according to international criteria [19]. All the patients were assumed to be healthy. Only patients who signed the assent form after receiving a letter of information on the measurement protocol were included. Criteria for exclusion were pathologies involving hydration disorders: (a) heart failure, kidney failure, hepatocellular failure pregnancy, use of diuretics, corticoids or antidepressants, (b) any chronic pathology with a life expectancy <6 months, (c) patients with cancer given medication <6 months ago, any pathology preventing BIA (pacemaker, amputation of a limb), and (d) refusal of assent.

It is necessary to distinguish between the BF and BF% predictive equations involving a large number of populations, such as Lee et al. [12] on 5239 men and Heo et al. [13] on 6544 males, from those obtained from a number of individuals ranging from 139 to 2154 [9, 17–21]. All these studies involve simple anthropometric dimensions: weight (kg), stature (cm), BMI (Weight (kg)/stature^2^ (m) and waist circumference (cm) (Table 1). Total body fat estimates obtained by Lee et al. [12] and Heo et al. [13] have a coefficient of determination (R^2^) greater than 0.90 while the coefficient of determination in the other studies ranged from 0.79 to 0.88 (Table 1).

**Table 1:** Predictive estimation models of BF and BF% for men from anthropometric variables.

Authors	Predictive equations of BF (kg) and BF%	
Lee et al. [12] n = 5329	BF = -18.59–0.009 age + 0.226 Weight (kg)– 0.08 Stature (cm) + 0.387 waist circumference	R^2^ = 0.90
Lee et al. [12] n = 5329	BF% = 0.02–0.08 Weight (kg) - 0.07 Stature (cm) + 0.48 waist circumference	R^2^ = 0.73
Heo et al. [13] n = 3347	BF = -24.0 + 1.77 BMI	R^2^ = 0.92
Larsson et al. [14] n = 274	BF = 18.38 + 0.2572 Weight—0.1349 Stature (cm) + 0.457 waist circumference	R^2^ = 0.88
Heitmann et al. [15] n = 139	BF = 0.988 BMI +0.242 Weight (kg) + 0.094 age– 30.18	R^2^ = 0.89
Pasco et al. [20] n = 1299	BF% = -16.7 + 1.62 (BMI-mean) -0.06 (BMI-mean) ^2^ + 0.02 age—0,17 (BMI-mean) + 0.03 (BMI-mean) ^2^ + 0.04 age + 37.8	R^2^ = 0.83
Durenberg et al. [21] n = 1976	BF% = -11.4 +0.2 age + 1.294 BMI– 8	R^2^ = 0.88
Gallagher et al. [9] n = 1626	BF% = 64.5–848 (1/BMI) + 0.079 age -16.4 +0.05 age + 39 (1/BMI)	R^2^ = 0.86
Gomez-Ambrosi et al. [22] n = 2154	BF% = -44.988 + 0.503 age + 3.172 BMI—0.026 BMI^2^ - 0.02 BMI age + 0.00021 BMI^2^ age	R^2^ = 0.79

BF: Body fat mass measured by Dual-energy X-ray absorptiometry (DXA).

In order to test the mean and individual precision of the predictive equations, we carried out anthropometric measurements on 120 adult men aged 18 to 82 years whose BF and BF% were obtained by absorptiometry DXA. The anthropometric measurements collected are those used in the predictive equations of previous studies, weight (kg), stature (cm), BMI and waist circumference (cm). The weight is raised to the nearest 100g using a Tanita scale, the stature is measured with a steel height gauge to the nearest 5mm and the waist circumference is measured with a metric ribbon at the upper lateral edge of the hip crest to the nearest 0.1 cm. The study obtained approval from the French Centre National de la Recherche Scientifique (CNRS). All participants gave their oral and written consent to participate in the studies in accordance with the Helsinki Declaration.

The results of DXA absorptiometry were obtained with a Hologic QDR-4500 W (version 11.25, Hologic Bedford, Mass USA). The subject is lies down for 7 minutes and radiation exposure is very low. The subject’s weight is calculated with a precision of less than 1%. The body composition result is available immediately after each examination.

The comparison between the mean values and the standard deviation of the anthropometric variables of our results and those of previous work with a wide sampling was made with the Student’s “t” test. The Fisher test was used to compare variances. The estimates of BF and BF% on our sample using the equations in the literature were compared to the actual values of the DXA measurements in our sample. First, the equations of Lee et al. [12] and Heo et al. [13] were used. The estimates obtained with the equation of Lee et al. [12] were then compared with those obtained using the equation of Larsson et al. [14] as it is the only one with the same variables as Lee et al. [12] but on a smaller sample size (Table 1). However, Lee et al. [12] includes age, which Larsson et al. [14] do not. However, the partial age regression coefficient is very low (-0.009) and can be ignored for our comparison.

The standard error of estimate is the standard deviation between the estimated BF or BF% values and the actual values. It is expressed as: SEE = √ (Y-Y’)^2^/N where Y represents the actual values of BF or BF% and Y’ the estimated values where N represents the population. The standard error of estimate is also equal to: SEE = σ √1- R^2^ where σ represents the standard deviation of BF or BF%. In this case SEE corresponds to the standard deviation of the differences of fat measurements between the estimated values and the DXA values. For each test, p<0.05 is considered the significance threshold.

## Results

Individual data is presented in S1 Table. The mean values and standard deviations of anthropometric measurements from our study, as well as those of Lee et al. [12] and Heo et al. [13] are shown in Table 2. There is no significant difference between the anthropometric measurements of our sample and those of the Lee et al. [12] population, however the DXA results are different. There are significant differences in anthropometric and BF DXA measurements between our sample and the results obtained by Heo et al. [13].

**Table 2:** Mean values and standard deviations of anthropometric variables, BF DXA and BF% DXA of our sample and previous studies with a high sample size.

	This study n = 120	Lee et al. [12] n = 5239	Heo et al. [13] n = 3347	"t" 1 vs 2	"t" 1 vs3
	x ± σ	x ± σ	x ± σ	p	p
Age (year)	41.6 ± 17.7	42.7± 22.4	45.4 ± 16.5	0.59	0.01
Weight (kg)	82.0 ± 18.8	82.9 ± 22.4	88.3 ± 18.7	0.66	<0.01
Stature (cm)	175.2 ± 7.4	176.5 ± 10.9	177.5 ± 7.2	0.19	<0.01
BMI (kg/m^2^)	26.7 ± 5.8	26.6 ± 5.8	28.0 ± 5.5	0.85	0.01
Waist circ. (cm)	95.9 ± 16.8	95.8 ± 19.5	100.4± 19.5	0.95	0.01
BF DXA (kg)	18.6 ± 9.9	22.7 ± 11.8	26.0 ± 10.4	<0.01	<0.01
BF% DXA	21.4 ± 7.5	26.5 ± 8.0	-	<0.01	-

BF: Body fat mass measured by Dual-energy X-ray absorptiometry (DXA).

**Fig 1:**
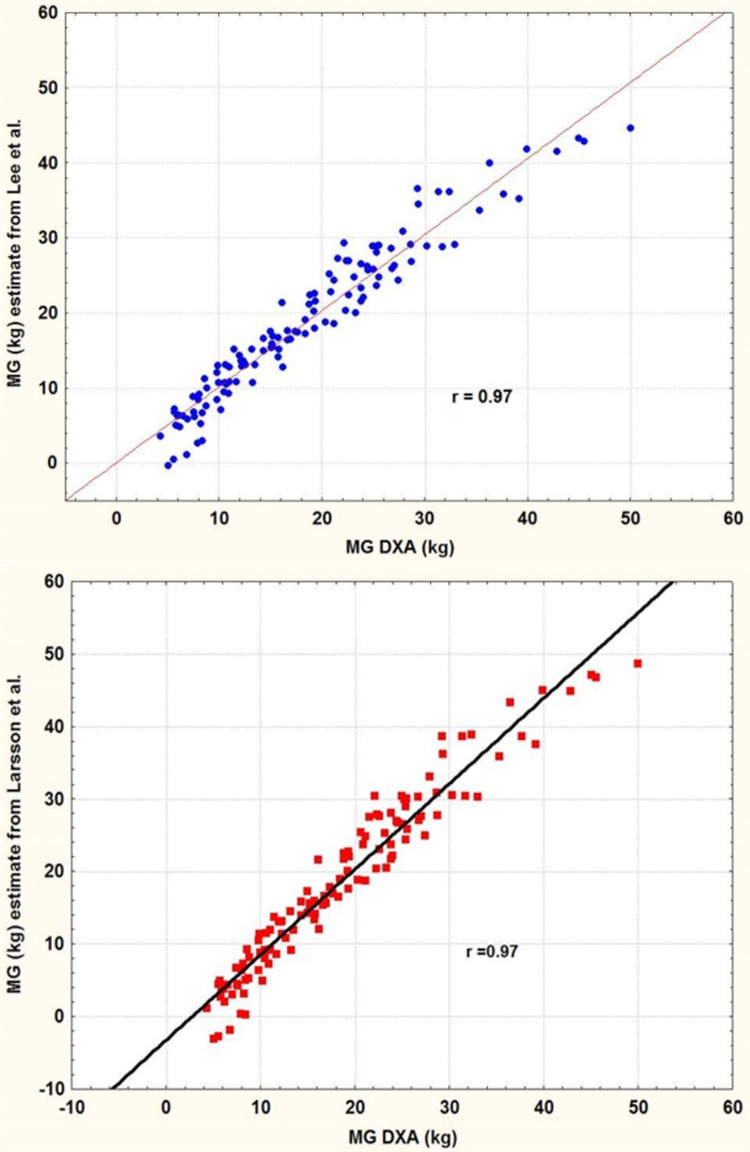
Regression between the BF DXA from our sample and the BF estimated using the equation of Lee et al. [12] (top) and Larsson et al. [14] (bottom).

**Fig 2:**
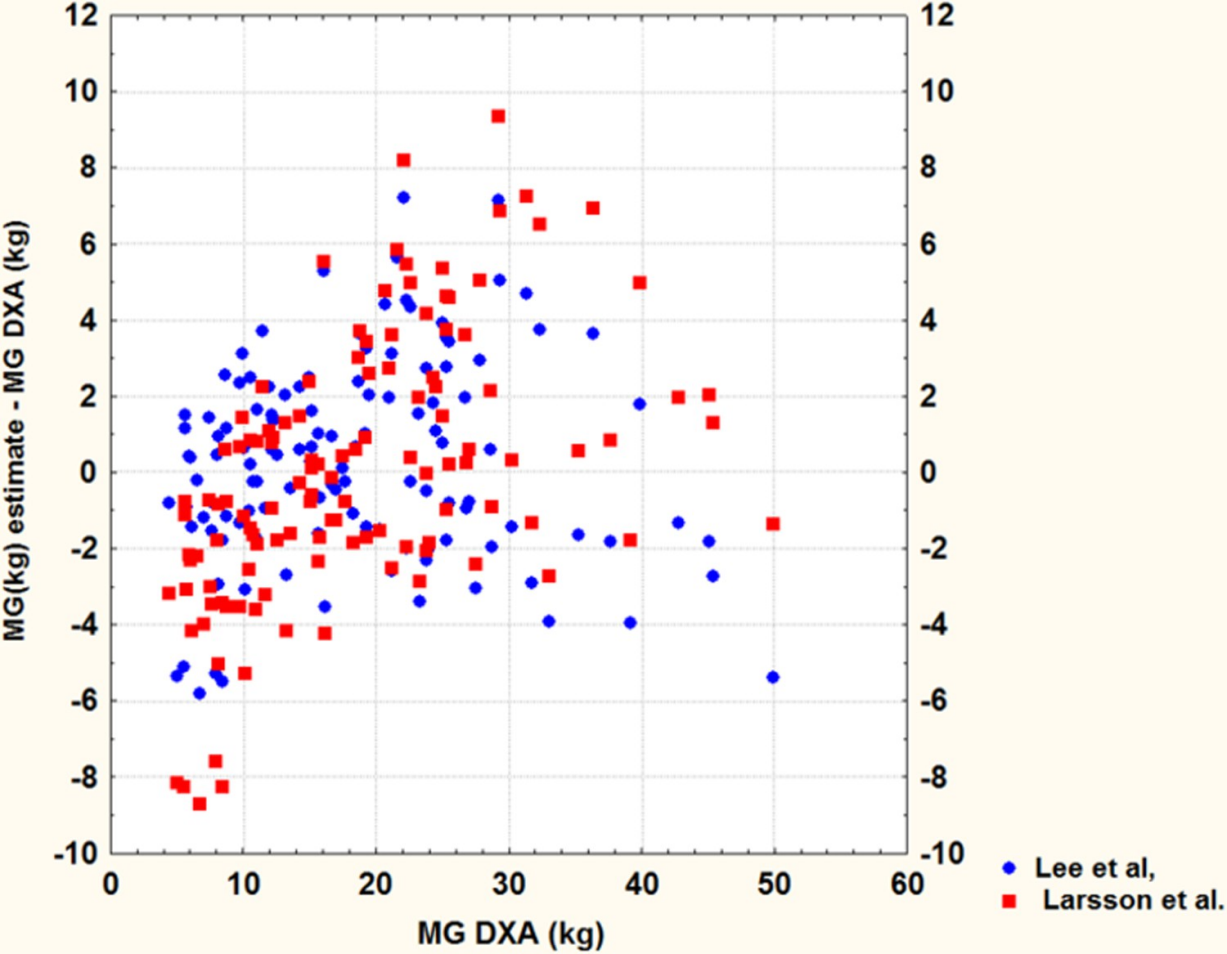
Individual deviations between BF estimates obtained from Lee et al.’s equation [12] and Larsson et al.’s equation [14] and the BF DXA of our sample.

The standard error of estimate (SEE) using different equations to that of our sample of 120 men is presented in Table 3. The SEE values reveal that there are no significant differences in the estimates obtained with equations A, B and C. Conversely, there is a significant difference between the DXA values and those obtained using Larsson et al. [14] equation (D). This difference is observed in the large dispersion of individual differences between estimates and actual BF values obtained by DXA (Fig 1). Consequently, the linear model proposed by Larsson et al. [14] cannot be applied to our sample because the dispersion of the deviations (SEE = 3.5 kg) is very high unlike that observed (SEE = 2.7) with the equations of Lee et al. [12] (Fig 2).

**Table 3:** Estimation of the BF and BF% using predictive equations on our sample.

	x ± σ	Mean difference	SEE*	t (*p*)	F	*p*	SEE^+^
BF (kg) PredEq A	22.7 ± 10.4	4.1	± 2.7	16.7 (<0.01)	1.12	NS	2.55
BF % PredEq B	27.2 ± 6.8	5.8	± 2.7	23.9 (<0.01)	1.08	NS	2.60
BF (kg) PredEq C	23.2 ± 10.4	4.6	± 2.9	17.1 (<0.01)	0	NS	2.90
BF (kg) PredEq D	22.8 ± 12.1	4.3	± 3.5	20.7 (<0.01)	1.52	< 0.05	2.84

PredEq: predictive equation. A and B: from Leet et al. [12], C: from Heo et al. [13], D: from Larsson et al. [17]. BF: Body fat mass measured by Dual-energy X-ray absorptiometry (DXA). SEE: Standard Error of Estimate. SEE*: for the population in this study (n = 120), SEE^+^: for the original study.

**Fig 3:**
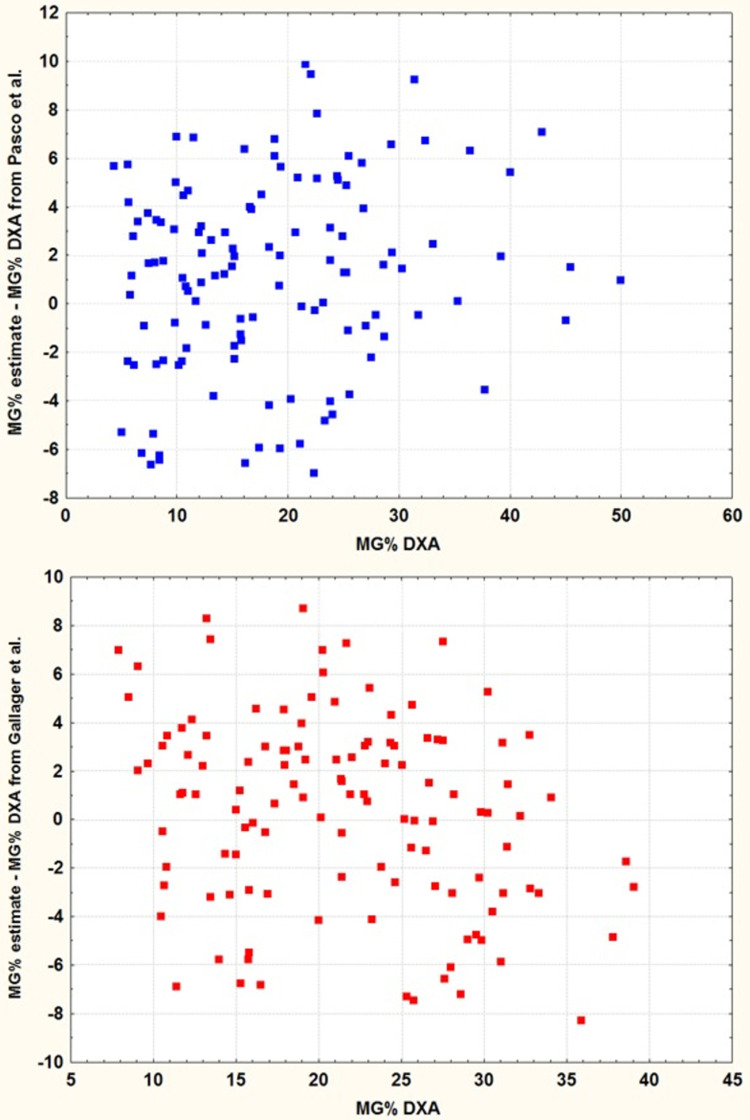
Individual deviations of BF% estimated with Pasco et al. [20] et Gallagher et al. [9] equations against BF% DXA. Estimated values show a high dispersion ± 10.

The predictive equations of Heitmann et al. [15] for BF and Pasco et al. [20], Durenberg et al. [21], Gallagher et al. [9] and Gomez-Ambrosi et al. [22] for BF% (Table 1) were also tested on our sample. The mean difference and SEE between the estimated values and the DXA values of our sample are grouped in Table 4. The SEE values between estimated BF% and BF% DXA are between 3.94 and 4.76 which reveal very large individual dispersions (Fig 3). In 95% of cases individual dispersions are included in the range 7.7 kg.

**Table 4:** Estimation of the BF and BF% from predictive equations compared with BF DXA and BF% DXA on our sample.

Auteurs	N	Mean difference ± SEE*	SEE^+^	F	*p*
Heitmann et al. [15]	93	6.8 ± 4.73	3.3	2.0	< 0.05
Pasco et al. [20]	1299	1.1 ± 3.95	4.0	1.0	NS
Durenberg et al. [21]	1976	2.0 ± 4.76	2.5	3.6	< 0.05
Gallager et al. [9]	613	0.3 ± 3.94	4.0	1.0	NS
Gomez-Ambrosi et al. [22]	2154	4.3 ± 4.17	4.7	1.3	< 0.05

BF: Body fat mass measured by Dual-energy X-ray absorptiometry (DXA). SEE: Standard Error of Estimate. SEE*: for the population in this study (n = 120), SEE^+^: for the original study.

The predictive equations of BF and BF% developed by Lee et al. [12] and Heo et al. [13] were applied to our sample. The mean values of the BF and BF% estimates, as well as the mean differences between the estimated BF and BF% values and the BF DXA and BF% DXA values are presented in Table 3. The BF estimates based on the equations of Lee et al. [12] and Heo et al. [13] show a significant overestimation of mean values but no significant difference in variances (Table 3). In order to correct this overestimation, BF and BF% were obtained in five subjects with a BMI (26.3 ≤ BMI ≤ 27.0) close to the average BMI of Lee et al. [12] which is 26.6 kg/m^2^. The BF DXA of these five individuals is 18.0 kg. Using equation A [12], we obtain a BF of 22.2 kg for these five individuals, which corresponds to an overestimation of 4.2 kg. A correction factor of -4.2 kg in equation A has to be included to readjust the estimated BF mean. Similarly, with the same five subjects, we obtained an estimate of BF% DXA (equation B) [12] equal to 28% while the direct measurement of BF% DXA gives 22.15%, an overestimate of 5.85%. Therefore, an adjustment factor of -5.85 must be introduced into equation B to readjust the estimated BF% values. Equation C [13] which includes BMI only gives a prediction of BF equal to 23.2 kg instead of 18.6 kg, an overestimation of 4.6 kg. This average overestimation can be corrected using the same process we used to correct the equations of Lee et al. [12].

## Discussion

In this study we have assessed the predictive equations of BF and BF% to obtain a cross-validation between the anthropometric measurement and DXA values. The interest of our study is to show that we can use the predictive equations of BF and BF% with a good accuracy from anthropometric measurements on different samples or populations. However, the predictive equations used in the scientific literature may present different estimates on both mean and individual values. For these reasons, we tested different predictive equations of BF and BF% obtained on different populations and we analyzed and interpreted these differences at the individual level.

### Similar studies

The anthropometric characteristics in our sample are indeed comparable to those of the individuals measured by Lee et al. [12]. There are significant differences between the mean values of BF DXA and BF% DXA and the results obtained using Lee et al.’s equation [12] despite the fact that DXA absorptiometry measurements are produced by a similar Hologic QDR 4500 device. When applied to our sample the BF predictive equation of Lee et al. [12] gives a significantly overestimated mean value relative to the exact measurement of BF DXA. Starting from similar anthropometric data between our sample and that of Lee et al. the DXA values which are dependent on the DXA machine show significant differences suggesting differences between the DXA devices. The average shift using the Lee equation on our population goes in the same direction. The average overestimation using the Lee et al. equation has to be calibrated by introducing a correction factor. The corrective term can be obtained by estimating BF and BF% on a limited number of subjects whose BMI is comparable to the average BMI of the population used to build the equation. This corrective term, of -4.2 kg for BF or 5.85% for BF% in the equations of Lee et al. [12], makes it possible to carry out a translation 239-243of all values. Under these conditions, the individual deviations completely overlap with those obtained in our sample by DXA. The SEE obtained from the equation of Lee et al. [12] on our sample (2.7) is low and equivalent to that obtained by these authors on their own sample, 2.55. Therefore, the overestimation using Lee et al.’s equation on our sample is the result of using different DXA devices which needs to be corrected.

### Different studies

Among other works which have proposed predictive equations, we selected those whose BF and BF% predictive equations involving the same anthropometric measurements as ours (Table 1). Despite a wide variety of sample size, the differences between the BF and BF% estimates and BF DXA and BF% DXA values present a dispersion ranging from 3.94 to 4.76. This therefore exceeds 3.5, the limit suggested by Lohman et al. [23] for the method to be of acceptable accuracy so that it can be used. The predictive equation of Larsson et al. [14] applied to the BF shows a dispersion of deviations of 3.5 for our sample despite the introduction of a correction factor (Table 3). This dispersion is significantly higher than that observed in the original analysis (SEE = 2.84). The equation proposed by Larsson et al. [14] is therefore not applicable to our sample. The large dispersions resulting from the application of all these equations are likely the result of insufficient variability in anthropometric measurements collected in the different cohorts. In the same way, we note that the predictive equations of BF and BF% of Heitman [15], Pasco [20], Durenberg [21], Gallager [9] and Gomez-Ambrosi [22] present values of a standard error of estimate (SEE) greater than 3.5 which does not allow to obtain a good precision at the individual level. Consequently, BF and BF% estimate from their predictive equations are too imprecise versus the DXA measurements obtained with our sample.

### Strengths and limitations

As already suggested [e.g. 11], our study shows that the use of predictive BF and BF% equations derived from anthropometric measurements are applicable to different samples. However, our work reveals two very important points to take into account when extrapolating the use of predictive equations. First of all, we suggest that some formulae only produce estimates with SEE exceeding 3, in which case the dispersion of the estimated values in relation to the real ones becomes very high and therefore their use is not acceptable. Only equations based on a large number of individuals allow estimates of values with SEE less than 3. Because of the large sample size these equations would have a sufficiently large anthropometric measurement variability in the different cohorts to be able to include the diversity of anthropometric values for the same body fat value. Secondly, it should be kept in mind that differences in software and in the scanning speed of DXA measuring instruments can lead to a shift of average values.

## Conclusion

The extrapolation of predictive equations should be accompanied by some DXA direct measurements in the population under study. This would confirm that 1) the equation does not produce an average shift and, if this is the case, 2) to allow the calculation of the correction factor. We have shown that this correction can be made by calculating a corrective term obtained from BF DXA values of subjects with anthropometric measurements similar to the mean of the reference population whose predictive equation is used.

## Supporting information

S1 Table Individual data. (PDF)
