# Supplementary material for: Measuring body fat—How accurate is the extrapolation of predictive models in epidemiology?
Source: PLoS One. 2022 Feb 10;17(2):e0263590. doi: 10.1371/journal.pone.0263590 (PMC8830724; doi:10.1371/journal.pone.0263590)
Supplement: S1 Table — (PDF) [file pone.0263590.s001.pdf]

S1 Table: Individual data

Subject	Age (year)	Weight (kg)	Stature (cm)	BMI	Waist circ (cm)	BF DXA (kg)	BF% DXA
1.0	30.0	44.2	161.0	17.1	65.5	5.0	11.4
2.0	58.0	48.8	169.0	17.1	68.0	6.8	14.0
3.0	41.0	52.0	171.0	17.8	65.0	5.5	10.7
4.0	55.0	53.5	173.0	17.9	71.0	8.4	15.7
5.0	43.0	50.0	167.0	17.9	80.0	7.7	15.3
6.0	22.0	59.0	180.0	18.2	74.0	6.2	10.5
7.0	57.0	61.0	181.0	18.6	77.0	5.9	9.7
8.0	36.0	50.0	162.0	19.1	70.0	7.9	15.8
9.0	29.0	51.0	163.0	19.2	80.0	8.4	16.5
10.0	22.0	65.0	184.0	19.2	74.0	7.0	10.8
11.0	67.0	62.0	178.0	19.6	78.0	5.6	9.1
12.0	23.0	55.0	167.0	19.7	74.0	5.8	10.5
13.0	23.0	65.0	181.0	19.8	78.0	8.8	13.5
14.0	53.0	55.0	165.0	20.2	70.0	4.3	7.9
15.0	23.0	67.0	182.0	20.2	74.0	6.1	9.1
16.0	25.0	56.0	166.0	20.3	74.0	8.2	14.6
17.0	28.0	64.2	175.5	20.8	76.0	10.2	15.8
18.0	52.0	65.0	176.0	21.0	81.0	10.9	16.8
19.0	25.0	63.7	174.0	21.0	75.0	7.5	11.8
20.0	23.0	69.0	181.0	21.1	78.0	8.0	11.7
21.0	24.0	68.5	180.0	21.1	78.0	9.8	14.3
22.0	34.0	59.8	168.0	21.2	75.0	6.5	10.9
23.0	48.0	77.0	190.0	21.3	81.0	11.7	15.2
24.0	25.0	84.0	198.0	21.4	84.5	12.6	15.0
25.0	39.0	73.5	185.0	21.5	83.0	8.6	11.7
26.0	23.0	66.0	175.0	21.6	74.0	5.6	8.5
27.0	23.0	70.0	180.0	21.6	78.0	7.4	10.6
28.0	23.0	70.0	180.0	21.6	81.0	8.8	12.6
29.0	23.0	62.0	169.0	21.7	82.0	10.5	16.9
30.0	66.0	64.2	171.0	22.0	84.0	10.8	16.8
31.0	22.0	67.0	173.0	22.4	79.0	8.1	12.1
32.0	29.0	66.5	172.0	22.5	83.0	13.3	20.0
33.0	24.0	70.0	176.0	22.6	82.0	10.5	15.0
34.0	22.0	75.0	182.0	22.6	84.0	9.8	13.0
35.0	22.0	71.0	177.0	22.7	82.0	11.1	15.6
36.0	82.0	71.0	177.0	22.7	94.0	15.2	21.4
37.0	80.0	74.0	180.0	22.8	92.0	15.8	21.4
38.0	21.0	76.0	181.0	23.2	85.0	12.2	16.1
39.0	23.0	80.0	185.0	23.4	84.0	9.9	12.3
40.0	22.0	80.0	184.0	23.6	84.0	10.6	13.3
41.0	47.0	78.0	181.0	23.8	94.0	15.7	20.1
42.0	57.0	75.5	178.0	23.8	79.0	10.0	13.2
43.0	38.0	79.0	182.0	23.8	95.0	18.3	23.2
44.0	80.0	62.8	162.0	23.9	91.0	12.3	19.6
45.0	29.0	76.0	178.0	24.0	88.0	12.0	15.7
46.0	36.0	71.0	172.0	24.0	94.0	15.2	21.4
47.0	27.0	64.0	163.0	24.1	88.0	16.2	25.3

48.0	42.0	73.0	174.0	24.1	86.0	13.5	18.5
49.0	71.0	69.0	169.0	24.2	90.0	15.7	22.8
50.0	80.0	62.0	160.0	24.2	101.0	17.4	28.1
51.0	47.0	85.0	187.0	24.3	87.0	11.4	13.5
52.0	58.0	68.0	167.0	24.4	89.0	12.2	17.9
53.0	21.0	88.0	189.0	24.6	90.0	15.2	17.3
54.0	43.0	73.0	172.0	24.7	91.0	13.2	18.0
55.0	50.0	80.0	180.0	24.7	89.0	15.0	18.8
56.0	35.0	75.0	174.0	24.8	89.5	14.3	19.1
57.0	40.0	79.0	178.0	24.9	92.0	16.9	21.4
58.0	39.0	80.0	179.0	25.0	92.0	14.3	17.9
59.0	39.0	68.0	165.0	25.0	86.0	11.0	16.2
60.0	19.0	75.0	173.0	25.1	97.0	19.3	25.7
61.0	56.0	71.0	167.0	25.5	97.0	15.0	21.1
62.0	39.0	78.0	175.0	25.5	102.0	22.3	28.6
63.0	60.0	75.0	171.0	25.6	99.0	20.3	27.1
64.0	56.0	73.0	168.0	25.9	99.0	21.2	29.0
65.0	22.0	93.0	188.0	26.3	89.0	16.7	18.0
66.0	47.0	84.0	178.0	26.5	99.0	19.2	22.9
67.0	27.0	86.6	179.0	27.0	87.5	16.6	19.2
68.0	62.0	80.0	172.0	27.0	97.0	18.4	23.0
69.0	21.0	87.0	179.0	27.2	102.0	24.0	27.6
70.0	36.0	88.0	180.0	27.2	103.0	19.3	21.9
71.0	56.0	84.0	175.0	27.4	91.0	17.6	21.0
72.0	46.0	94.0	185.0	27.5	107.0	20.7	22.0
73.0	25.0	89.0	180.0	27.5	107.0	21.2	23.8
74.0	51.0	92.0	183.0	27.5	107.0	23.2	25.2
75.0	23.0	85.0	175.0	27.8	101.0	23.8	28.0
76.0	21.0	91.0	180.0	28.1	112.5	22.4	24.6
77.0	66.0	82.0	170.0	28.4	110.0	25.5	31.1
78.0	59.0	93.0	181.0	28.4	100.0	18.8	20.2
79.0	42.0	75.0	162.0	28.6	100.0	23.3	31.1
80.0	23.0	85.0	172.0	28.7	100.0	16.1	18.9
81.0	62.0	93.8	180.0	29.0	108.0	25.0	26.7
82.0	79.0	84.0	170.0	29.1	106.0	25.4	30.2
83.0	41.0	98.0	183.0	29.3	114.0	25.3	25.8
84.0	28.0	93.0	176.0	30.0	104.0	27.4	29.5
85.0	72.0	92.0	175.0	30.0	116.0	25.0	27.2
86.0	56.0	81.2	164.0	30.2	100.0	18.8	23.1
87.0	49.0	113.0	193.0	30.3	103.0	21.6	19.1
88.0	23.0	93.0	175.0	30.4	101.0	23.8	25.6
89.0	58.0	104.0	185.0	30.4	106.0	22.6	21.7
90.0	54.0	100.0	180.5	30.7	105.5	24.4	24.4
91.0	65.0	86.0	167.0	30.8	111.0	27.0	31.4
92.0	31.0	85.0	166.0	30.8	99.0	19.4	22.8
93.0	43.0	103.0	182.0	31.1	109.0	25.3	24.6
94.0	64.0	88.0	168.0	31.2	100.0	22.6	25.7
95.0	41.0	86.0	166.0	31.2	102.0	20.9	24.3
96.0	22.0	111.0	188.0	31.4	122.0	29.4	26.5
97.0	19.0	96.0	174.5	31.5	108.0	28.7	29.9

98.0	56.0	96.0	173.0	32.1	114.0	28.6	29.8
99.0	26.0	109.0	184.0	32.2	109.0	22.1	20.3
100.0	29.0	88.5	165.5	32.3	110.0	23.8	26.9
101.0	28.0	91.4	168.0	32.4	120.0	27.9	30.5
102.0	52.0	92.0	168.0	32.6	106.0	24.5	26.6
103.0	23.0	106.0	180.0	32.7	109.0	25.5	24.1
104.0	47.0	96.6	170.0	33.4	112.0	31.7	32.8
105.0	40.0	95.0	168.0	33.7	105.0	26.8	28.2
106.0	64.0	94.0	167.0	33.7	113.0	30.3	32.2
107.0	22.0	117.0	185.0	34.2	123.0	29.3	25.0
108.0	48.0	97.0	168.0	34.4	111.0	26.7	27.5
109.0	42.0	106.0	174.5	34.8	120.0	35.3	33.3
110.0	18.0	111.0	178.0	35.0	106.0	33.0	29.7
111.0	20.0	105.0	173.0	35.1	126.0	37.7	35.9
112.0	75.0	115.0	180.0	35.5	120.0	39.2	34.1
113.0	74.0	107.0	171.0	36.6	125.0	32.4	30.3
114.0	68.0	114.0	176.0	36.8	122.0	31.4	27.5
115.0	55.0	117.0	174.0	38.6	130.0	36.4	31.1
116.0	44.0	127.0	181.0	38.8	130.0	40.0	31.5
117.0	42.0	119.0	174.0	39.3	137.0	45.0	37.8
118.0	56.0	131.0	176.0	42.3	126.0	42.9	32.7
119.0	61.0	118.0	167.0	42.3	135.0	45.5	38.6
120.0	54.0	128.0	172.5	43.0	135.0	50.0	39.1

Waist circ: waist circumference, BF: Body fat mass measured by Dual-energy X-ray absorptiometry (DXA)
